# Epidemiology of Road Traffic Incidents in Peru 1973–2008: Incidence, Mortality, and Fatality

**DOI:** 10.1371/journal.pone.0099662

**Published:** 2014-06-13

**Authors:** J. Jaime Miranda, Luis A. López-Rivera, D. Alex Quistberg, Edmundo Rosales-Mayor, Camila Gianella, Ada Paca-Palao, Diego Luna, Luis Huicho, PIAT Working Group, PIAT Working Group, Ada Paca, López Luis, Diego Luna, Edmundo Rosales, Pablo Best, Pablo Best, Miriam Egúsquiza, Camila Gianella, Claudia Lema, Esperanza Ludeña, J. Jaime Miranda, Luis Huicho

**Affiliations:** 1 Programa de Investigación en Accidentes de Tránsito, Salud Sin Límites Perú, Lima, Peru; 2 CRONICAS, Center of Excellence in Chronic Diseases, Universidad Peruana Cayetano Heredia, Lima, Peru; 3 School of Medicine, Universidad Peruana Cayetano Heredia, Lima, Peru; 4 EDHUCASALUD, Asociación Civil para la Educación en Derechos Humanos con Aplicación en Salud, Lima, Peru; 5 Programa Nacional de Empleo Juvenil Jóvenes a la Obra, Ministerio del Trabajo y Promoción del Empleo, Lima, Peru; 6 Department of Pediatrics, School of Medicine, University of Washington, Seattle, Washington, United States of America; 7 Harborview Injury Prevention & Research Center, University of Washington. Seattle, Washington, United States of America; 8 Centro de Trastornos Respiratorios del Sueño (CENTRES), Clínica Anglo Americana, Lima, Peru; 9 Grupo de Investigación en Sueño (GIS), Lima, Peru; 10 Hospital Clínic de Barcelona, Barcelona, Spain; 11 Departamento de Ciencias Sociales y Políticas, Universidad del Pacífico, Lima, Peru; 12 Asociación Civil “Gobierno Coherente”, Lima, Peru; 13 Department of Pediatrics, Instituto Nacional de Salud del Niño, Lima, Peru; 14 School of Medicine, Universidad Nacional Mayor de San Marcos, Lima, Peru; Indiana University and Moi University, United States of America

## Abstract

**Background:**

The epidemiological profile and trends of road traffic injuries (RTIs) in Peru have not been well-defined, though this is a necessary step to address this significant public health problem in Peru. The objective of this study was to determine trends of incidence, mortality, and fatality of RTIs in Peru during 1973–2008, as well as their relationship to population trends such as economic growth.

**Methods and Findings:**

Secondary aggregated databases were used to estimate incidence, mortality and fatality rate ratios (IRRs) of RTIs. These estimates were standardized to age groups and sex of the 2008 Peruvian population. Negative binomial regression and cubic spline curves were used for multivariable analysis. During the 35-year period there were 952,668 road traffic victims, injured or killed. The adjusted yearly incidence of RTIs increased by 3.59 (95% CI 2.43–5.31) on average. We did not observe any significant trends in the yearly mortality rate. The total adjusted yearly fatality rate decreased by 0.26 (95% CI 0.15–0.43), while among adults the fatality rate increased by 1.25 (95% CI 1.09–1.43). Models fitted with splines suggest that the incidence follows a bimodal curve and closely followed trends in the gross domestic product (GDP) per capita

**Conclusions:**

The significant increasing incidence of RTIs in Peru affirms their growing threat to public health. A substantial improvement of information systems for RTIs is needed to create a more accurate epidemiologic profile of RTIs in Peru. This approach can be of use in other similar low and middle-income settings to inform about the local challenges posed by RTIs.

## Introduction

Road traffic incidents (RTIs) constitute a worldwide public health problem [1]–[3] and have been identified as a public health research priority in Peru [4]. In Peru, with few exceptions [5]–[9], there are no systematic studies of the epidemiology of RTIs and their trends. This contributes to the existence of an out-of-date, inadequately defined epidemiological profile. While a detailed epidemiology of RTIs for all of Peru is not available, international reports indicate that there are over 3,000 road fatalities annually in Peru and that road trauma is the fourth leading cause of premature death leading to an estimated 172,000 years of life lost in 2010 [10], [11]. One of the major limitations to addressing this issue is the lack of a suitable informatics system [12], though there are recent efforts to establish a hospital-based surveillance system [13]. A well-defined epidemiology of RTIs is a critical tool to develop prevention and control strategies based on solid evidence [12].

Having up-to-date and accurate health statistics is important not only to develop and implement good policies, but also for ensuring that developmental and health goals are being met [14]–[16]. Concrete health statistics also improve the ability to determine needs and resource allocation priorities, as well as the assessment and evaluation of projects and interventions [17]–[18] and long-term trends [19]–[22]. These statistics include the collection of both indicators and contributing factors. It is also important to be able to collect health statistics within subgroups that may be at higher risk for the disease of interest when possible, in order to monitor and assess trends in these groups [14].

Using available data sources in Peru [23], we evaluated the incidence, mortality and fatality trends of RTIs in Peru from 1973 through 2008 to assess the utility and challenges of these data for developing the epidemiological profile of RTIs in Peru over recent decades. We also assessed the effects of population growth, vehicle density, road density, and economic growth on road traffic injury trends which have been demonstrated to be associated with road trauma in other countries [24], [25].

## Methods

### Data Sources

We had direct access to data from the *Estado Mayor de la Policía Nacional del Perú* (PNP, Peruvian National Police) to identify road traffic injuries from motor vehicle collisions from 1973 to 2008. The PNP are responsible for reporting and recording motor vehicle collisions. Each commissary is responsible for reporting its local data, which is reported in aggregate annually to regional and national administrative units of the PNP. Local commissary records for the period of study are maintained on paper records in collision log notebooks. We defined victims as those who were injured or killed by road traffic incident, which could include motorists, passengers, pedestrians, cyclists or other road users. The police may report the number injured, but these data are not linked to actual medical conditions or outcomes. “Injured,” thus, could range from very light injuries to extremely severe. Reporting may vary between individual police officers and commissaries nationwide, but there are no studies to determine this variability nationally or temporally and is a limitation of these data.

### Consistency of the data

**Figure 1:**
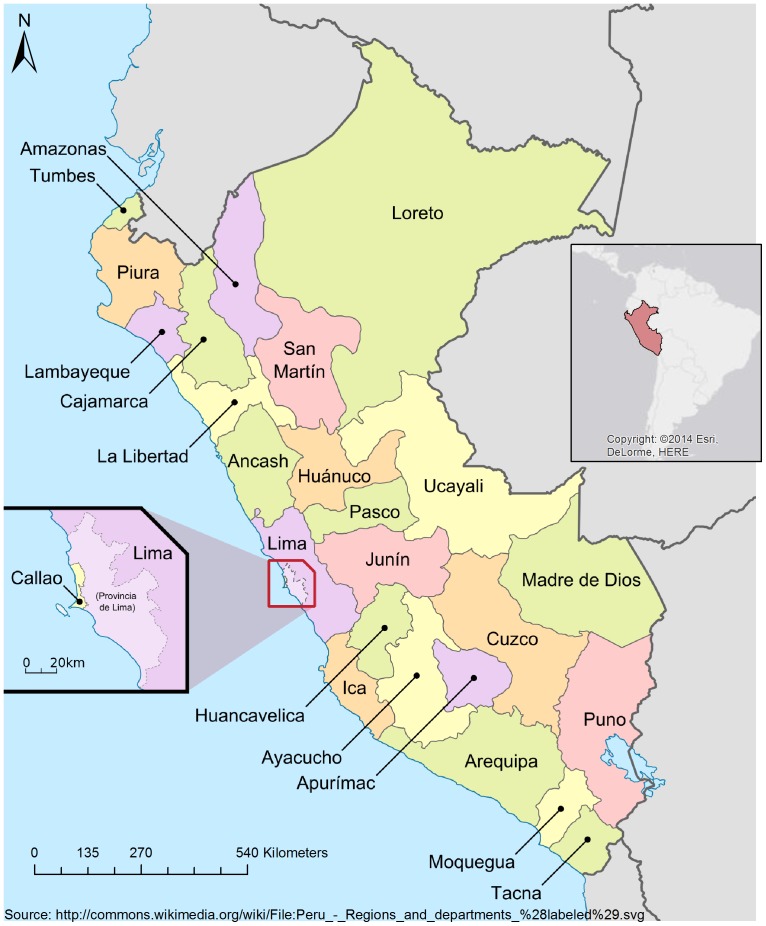
Map of the regions of Peru.

From 1973 to 1992 data are yearly aggregated totals of all victims of RTIs nationally and by Peru's 25 regions (administrative divisions of Peru locally known as departments, Figure 1) and the province of Lima. From 1993 to 1999, data are aggregated totals of injured and fatal victims of RTIs. From 2000 to 2008, data are aggregated totals of injured and fatal victims by age (less than 18 years or 18 years and older) and sex. To complete the PNP outcome data on injury or fatality from 1973 to 1992, we included statistics compiled from aggregated data published by the *Instituto Nacional de Estadística e Informática* (INEI) and by the *Centro de Investigación y de Asesoría del Transporte Terrestre* [26]–[28]. Both institutions used the data provided by the PNP as the basis for data disaggregated into outcome during this period. To provide data not collected on age and gender for 1973–1999 we used data from 2000–2008. This strategy was based on the observation that the proportional mortality for each of these categories was relatively constant from 2000 to 2008, thus we assumed similar proportional consistency in the previous period, to weight the total outcomes by age and sex for the period of 1973–1999. The weights were determined by assessing the mean for each subpopulation from 2000 to 2008 which was then divided by the sum of subpopulation means. To obtain the total subpopulation count, the total number of deaths or injuries was then multiplied by this weight.

### Study Variables

#### Incidence

Yearly incidence was calculated as the number of persons dead or injured (the total affected population) divided by the total susceptible population per 100,000 inhabitants. The susceptible population was defined as the estimated total population during the month of June for each year of the study, as reported by the INEI [29], [30]. For the period of 1993–2008, given the availability of the data, the incidence calculations were carried out at the provincial level, using the provincial populations by year as reported by the INEI [29], [31], [32].

#### Mortality

Yearly mortality was calculated by dividing total number of deaths by the total susceptible population per 100,000 inhabitants. The information collected by the PNP is based only on what occurs at the scene of the road traffic incident, and thus is biased towards an underreporting of the mortality given that it does not generally include deaths that occur after the victims leave the scene of the incident. These deaths are not assigned any International Classification and Related Health Problems (ICD) revisions 9 or 10 codes by the police. The recent “Global Status Report on Road Safety” [1] published by the World Health Organization (WHO), following the recommendation of the European Conference of Ministers of Transport [33], recommends that the definition of road traffic deaths should include deaths that occur within 30 days after the incident. They suggest therefore correcting mortality estimates by a factor of 1.30 [1], [33] for those registries that collect only information on deaths occurring on the incident scene or the first day after the incident, such as the data of the PNP. This adjustment factor has been used also by the Economic Commission for Latin America and the Caribbean (ECLAC) [34].

#### Fatality

Yearly fatality was calculated by dividing the number of deaths by the number of persons involved in RTIs, and expressing it as a percentage. For the period of 1993 to 2008, given the availability of the data, it was also calculated for regions, using the regional population by year as provided by the INEI [29], [31], [32].

### Statistical Analysis

We calculated each indicator for the total population, by sex, and by age group. Frequencies and proportions are reported in the descriptive analysis.

#### Contextual Factors

Many factors can influence the occurrence of RTIs. Because of this and due to the ecological nature of this study, we adjusted the crude incidence, mortality and fatality rates by total exposed population, vehicle density (parking lots per each 1000 inhabitants) and road density (paved highways per surface area of the entire country), generated from secondary sources of information [35], [36].

We additionally adjusted incidence rates by gross domestic product (GDP) per capita for the study period, using 1994 as the reference year [37]. This adjustment is determined by the *Banco Central de Reserva del Perú* (Peruvian Central Reserve Bank) and allows GDP comparison across various years. We did not adjust for poverty level because of its changing definition over time [32], and because of absence of data before 1990.

#### Trend Analysis

In order to estimate the trends of the indicators of interest for the study period we considered several different potential statistical modeling techniques. Given we could not assume a linear or normal distribution of the events of interest because we used count-type data, we explored recommended, alternative regression methods to evaluate temporal trends, including Poisson and negative binomial regression [38]. Both these methods have been widely used under other conditions [39], [40] and also for the analysis of RTIs [41]. In the current study, after a detailed evaluation, we determined that a negative binomial regression function would better fit and describe the data than Poisson regression. Using negative binomial regression we obtained incidence rate ratios (IRR) [42] and 95% confidence intervals (95% CI). The IRR obtained can be interpreted as the change in the indicator or its subcategories per one year increase. We progressively evaluated the contextual factors of interest, adding each one and determining if the new model better described the analyzed data. Likelihood ratio tests were used to compare models to evaluate model fit. We report crude and adjusted estimates. The covariates we evaluated are total population, vehicle density, road density, allowing the importation of used vehicles, implementation of Seguro Obligatorio contra Accidentes de Transito [Obligatory Insurance for Road Accidents] (SOAT), and implementation of *Plan Tolerancia Cero* [Zero Tolerance Plan]. SOAT is a national insurance plan that all registered vehicles are required to have (since 2002) in order to pay for some of the health care costs for any person injured in a motor vehicle collision that the vehicle is involved in. The Zero Tolerance Plan is a national law passed in 2006 targeting public transportation and cargo transportation vehicles meant to ensure safe operation through technical revision of vehicles and assessing the driver's condition for operating the vehicle (e.g., driving under the influence of alcohol).

To evaluate the indicators by sex and age groups, each study variable was standardized to the 2008 Peruvian population distribution [31] through direct methods with established techniques [43].

#### Splines

Though the IRR provides an easily interpretable estimate, the distribution of the observed data over time could have a more complex, curvilinear form than the linear form assumed with the IRR. We conducted a visual exploration of the behavior of the data with cubic smoothing spline curves [44]. Splines were used to test for any complex curvilinear relationships. We manually determined the slope by testing distinct numbers of knots in the curves (3, 4, 5, etc.). Normal, Poisson, and negative binomial distributions were tested for comparison. The final splines models were based on the observed “best fit” of the data, testing fit with quantile-quantile plots, in order to be sure we were using the best distribution. The Bayesian information criterion (BIC) was used to identify the model that best met parsimonious criteria, thus the model with the lowest BIC was selected [45].

Once the crude relationship between the dependent (incidence, mortality, and fatality) and independent (year) variables was tested, an *a priori* model adjusted by contextual factors was established. This adjusted model included total population, road density and vehicle density. For comparison purposes we also calculated and graphed GDP spline curves in the overall indicators graphics.

Statistical analysis was carried out using Stata 10 (STATA Corp, College Station, Texas, USA)

## Ethical Aspects

The study protocol was reviewed and approved by the Committee for Ethical Investigations of the *Instituto Nacional de Salud del Perú* (Peruvian National Institute of Health). The committee waived the need for consent for the use of the data.

## Results

Between 1973 and 2008 there were 952,668 persons affected by RTIs, which includes both dead and injured (Table 1). The incidence, mortality, and fatality rates, both crude and standardized for each year of the study, are presented in Tables 2, 3, and 4, respectively. Incidence rates by five-year periods are reported in Table 5.

**Table 1:** Absolute numbers of those reported injured or killed by RTIs in Peru, 1973–2008.

Year	Total Population	≥18 years*	<18 years*	Men Affected*	Women Affected*	Injured*	Dead*	Total Affected
1973	14,348,083	13,229	5,720	12,998	5,951	16,902	2,047	**18,949**
1974	14,751,106	12,793	5,723	13,160	5,356	15,650	2,866	**18,516**
1975	15,161,146	13,526	5,393	13,605	5,314	16,927	1,992	**18,919**
1976	15,580,838	14,412	8,786	16,437	6,761	21,385	1,813	**23,198**
1977	16,010,976	14,087	9,253	16,651	6,689	21,540	1,800	**23,340**
1978	16,447,572	14,597	9,272	15,719	8,150	22,177	1,692	**23,869**
1979	16,886,631	15,025	10,000	17,012	8,013	23,038	1,987	**25,025**
1980	17,324,179	16,959	10,640	19,098	8,501	25,496	2,103	**27,599**
1981	17,759,934	16,840	10,161	18,272	8,729	24,786	2,215	**27,001**
1982	18,196,557	16,519	9,016	18,313	7,222	23,595	1,940	**25,535**
1983	18,634,464	14,356	7,553	15,314	6,595	19,344	2,565	**21,909**
1984	19,074,066	16,085	6,428	16,080	6,433	19,850	2,663	**22,513**
1985	19,515,785	14,430	5,715	14,312	5,833	17,377	2,768	**20,145**
1986	19,962,821	16,609	6,249	16,140	6,718	19,716	3,142	**22,858**
1987	20,414,886	16,999	6,180	16,320	6,859	20,264	2,915	**23,179**
1988	20,867,194	12,235	4,452	11,940	4,747	14,801	1,886	**16,687**
1989	21,314,933	8,705	3,346	8,516	3,535	10,873	1,178	**12,051**
1990	21,753,328	7,678	9,168	11,946	4,900	14,096	2,750	**16,846**
1991	22,179,595	12,701	4,040	12,264	4,477	13,991	2,750	**16,741**
1992	22,596,921	13,955	1,956	8,931	6,980	14,044	1,867	**15,911**
1993	23,009,480	15,035	4,377	15,171	4,241	16,835	2,577	**19,412**
1994	23,421,416	10,285	1,944	9,411	2,818	9,780	2,449	**12,229**
1995	23,836,867	14,876	2,768	13,136	4,508	14,201	3,443	**17,644**
1996	24,257,671	12,681	2,726	11,916	3,491	12,559	2,848	**15,407**
1997	24,681,045	25,034	5,714	23,452	7,296	27,532	3,216	**30,748**
1998	25,104,276	24,316	5,424	22,504	7,236	26,417	3,323	**29,740**
1999	25,524,613	27,546	7,246	25,717	9,075	31,578	3,214	**34,792**
2000	25,939,329	24,476	8,587	25,260	7,803	29,945	3,118	**33,063**
2001	26,346,840	24,570	6,385	21,788	9,167	27,747	3,208	**30,955**
2002	26,748,972	25,534	7,282	23,156	9,660	29,887	2,929	**32,816**
2003	27,148,101	28,126	7,400	25,285	10,241	32,670	2,856	**35,526**
2004	27,546,574	29,940	8,563	26,917	11,586	35,337	3,166	**38,503**
2005	27,946,774	34,583	9,231	31,161	12,653	40,512	3,302	**43,814**
2006	28,348,700	40,089	10,224	35,433	14,880	46,832	3,481	**50,313**
2007	28,750,770	42,668	10,699	37,862	15,505	49,857	3,510	**53,367**
2008	29,152,987	42,120	11,428	38,001	15,547	50,059	3,489	**53,548**
**TOTAL**	**-**	**703,619**	**249,049**	**679,198**	**273,470**	**857,600**	**95,068**	**952,668**

*For 1973–1992, supopulation totals for age, sex and outcome are extrapolated from 2008–2008. For 1993–1999, supopulation totals for age and sex are extrapolated from 2000–2008.

**Table 2:** Crude and standardized* incident rates of RTIs per 100,000 inhabitants by age group and sex, 1973–2008.

	Crude Incidence Rate					Standardized Incidence Rate			
Year	Total	Males	Females	≥18 years	<18 years	Males	Females	≥18 years	<18 years
1973	132.1	179.8	83.6	184.9	79.5	90.4	41.6	111.9	27.7
1974	125.5	177.0	73.2	173.4	77.6	89.0	36.4	104.9	27.0
1975	124.8	178.1	70.7	177.7	71.4	89.5	35.1	107.6	24.9
1976	148.9	209.4	87.5	183.6	113.7	105.2	43.5	111.1	39.6
1977	145.8	206.5	84.2	173.9	117.0	103.8	41.9	105.3	40.7
1978	145.1	189.8	99.8	174.7	114.6	95.4	49.7	105.7	39.9
1979	148.2	200.1	95.6	174.3	121.0	100.6	47.5	105.5	42.1
1980	159.3	219.0	98.8	190.7	126.2	110.1	49.2	115.4	44.0
1981	152.0	204.4	99.0	183.6	118.3	102.7	49.2	111.1	41.2
1982	140.3	200.0	79.9	174.5	103.3	100.5	39.8	105.6	36.0
1983	117.6	163.3	71.3	147.1	85.1	82.1	35.4	89.0	29.7
1984	118.0	167.5	67.9	159.8	71.4	84.2	33.8	96.7	24.9
1985	103.2	145.7	60.2	139.1	62.5	73.2	29.9	84.2	21.8
1986	114.5	160.7	67.7	155.5	67.3	80.8	33.7	94.1	23.5
1987	113.5	158.9	67.6	154.6	65.6	79.9	33.6	93.5	22.9
1988	80.0	113.7	45.8	108.1	46.6	57.2	22.8	65.4	16.2
1989	56.5	79.4	33.4	74.8	34.6	39.9	16.6	45.2	12.1
1990	77.4	109.2	45.3	64.1	93.8	54.9	22.6	38.8	32.7
1991	75.5	109.9	40.6	103.2	40.9	55.2	20.2	62.5	14.3
1992	70.4	78.6	62.2	110.4	19.6	39.5	30.9	66.8	6.8
1993	84.4	131.0	37.1	115.9	43.6	65.9	18.5	70.1	15.2
1994	52.2	79.9	24.2	77.2	19.2	40.1	12.1	46.7	6.7
1995	74.0	109.5	38.1	108.8	27.2	55.0	18.9	65.8	9.5
1996	63.5	97.6	29.0	90.3	26.7	49.1	14.4	54.6	9.3
1997	124.6	188.8	59.5	173.4	55.8	94.9	29.6	104.9	19.4
1998	118.5	178.2	58.0	163.9	52.8	89.6	28.9	99.2	18.4
1999	136.3	200.3	71.6	180.8	70.4	100.7	35.6	109.4	24.5
2000	127.5	193.6	60.5	156.6	83.3	97.3	30.1	94.8	29.0
2001	117.5	164.4	70.0	153.3	61.9	82.6	34.8	92.8	21.6
2002	122.7	172.1	72.7	155.5	70.5	86.5	36.1	94.1	24.6
2003	130.9	185.2	75.9	167.2	71.7	93.1	37.8	101.2	25.0
2004	139.8	194.3	84.6	173.8	83.0	97.7	42.1	105.2	28.9
2005	156.8	221.8	91.1	196.2	89.5	111.5	45.3	118.7	31.2
2006	177.5	248.6	105.6	222.3	99.2	125.0	52.5	134.5	34.5
2007	185.6	262.0	108.4	231.3	103.9	131.7	53.9	140.0	36.2
2008	183.7	259.4	107.2	223.3	111.0	130.4	53.3	135.1	38.7

*Note: The data for incidence rates by sex or age groups were standardized to the Peruvian population of 2008.

**Table 3:** Crude and standardized* mortality rates of RTIs in Peru per 100,000 inhabitants by sex and age group, 1973–2008.

	Crude Mortality					Standardized Mortalidad			
Year	Total	Males	Females	≥18 years	<18 years	Males	Females	≥18 years	<18 years
1973	14.3	28.8	8.1	30.9	6.2	14.5	4.0	18.7	2.2
1974	19.4	39.3	11.0	42.0	8.5	19.7	5.5	25.4	3.0
1975	13.1	26.6	7.5	28.3	5.8	13.4	3.7	17.1	2.0
1976	11.6	23.5	6.6	25.0	5.1	11.8	3.3	15.1	1.8
1977	11.2	22.7	6.4	24.0	5.0	11.4	3.2	14.5	1.7
1978	10.3	20.8	5.8	21.9	4.6	10.5	2.9	13.3	1.6
1979	11.8	23.8	6.7	24.9	5.3	12.0	3.3	15.1	1.8
1980	12.1	24.6	6.9	25.6	5.5	12.4	3.4	15.5	1.9
1981	12.5	25.2	7.1	26.1	5.7	12.7	3.5	15.8	2.0
1982	10.7	21.6	6.0	22.2	4.9	10.8	3.0	13.4	1.7
1983	13.8	27.9	7.8	28.4	6.3	14.0	3.9	17.2	2.2
1984	14.0	28.3	7.9	28.6	6.5	14.2	3.9	17.3	2.3
1985	14.2	28.7	8.0	28.9	6.6	14.4	4.0	17.5	2.3
1986	15.7	31.9	8.9	31.8	7.4	16.0	4.4	19.2	2.6
1987	14.3	28.9	8.1	28.7	6.8	14.5	4.0	17.4	2.4
1988	9.0	18.3	5.1	18.0	4.3	9.2	2.5	10.9	1.5
1989	5.5	11.2	3.1	10.9	2.7	5.6	1.6	6.6	0.9
1990	12.6	25.6	7.2	24.8	6.2	12.9	3.6	15.0	2.1
1991	12.4	25.1	7.0	24.2	6.1	12.6	3.5	14.6	2.1
1992	8.3	16.7	4.7	16.0	4.1	8.4	2.3	9.7	1.4
1993	11.2	22.7	6.4	21.5	5.6	11.4	3.2	13.0	2.0
1994	10.5	21.2	5.9	19.9	5.3	10.6	3.0	12.0	1.9
1995	14.4	29.2	8.2	27.2	7.4	14.7	4.1	16.5	2.6
1996	11.7	23.8	6.7	21.9	6.1	11.9	3.3	13.3	2.1
1997	13.0	26.4	7.4	24.1	6.9	13.3	3.7	14.6	2.4
1998	13.2	26.8	7.5	24.2	7.1	13.5	3.7	14.7	2.5
1999	12.6	25.5	7.1	22.8	6.8	12.8	3.6	13.8	2.4
2000	12.0	25.3	5.9	20.3	8.6	12.7	2.9	12.3	3.0
2001	12.2	23.7	7.9	21.1	7.6	11.9	3.9	12.8	2.6
2002	11.0	21.4	7.0	18.7	7.2	10.8	3.5	11.3	2.5
2003	10.5	21.6	5.7	18.6	5.7	10.8	2.8	11.2	2.0
2004	11.5	23.3	6.5	19.8	6.8	11.7	3.2	12.0	2.4
2005	11.8	23.9	6.7	20.7	6.3	12.0	3.3	12.5	2.2
2006	12.3	25.5	6.3	21.3	6.6	12.8	3.1	12.9	2.3
2007	12.2	24.6	7.1	21.0	6.7	12.3	3.5	12.7	2.3
2008	12.0	24.3	6.8	20.6	6.3	12.2	3.4	12.5	2.2

*Note: The data for mortality rates by sex or age groups were standardized to the Peruvian population of 2008.

**Table 4:** Crude and standardized* fatality rates of RTIs in Peru per 100,000 RTI victims by sex and age group, 1973–2008.

	Crude Fatality Rate					Standardized Fatality Rate			
Year	Total	Males	Females	≥18 years	<18 years	Males	Females	≥18 years	<18 years
1973	10.8	16	9.7	16.7	7.8	8.1	4.8	10.1	2.7
1974	15.5	22.2	15.1	24.2	11	11.2	7.5	14.7	3.8
1975	10.5	14.9	10.5	15.9	8.1	7.5	5.2	9.6	2.8
1976	7.8	11.2	7.6	13.6	4.5	5.6	3.8	8.2	1.6
1977	7.7	11	7.6	13.8	4.3	5.5	3.8	8.4	1.5
1978	7.1	11	5.8	12.5	4	5.5	2.9	7.6	1.4
1979	7.9	11.9	7	14.3	4.3	6	3.5	8.7	1.5
1980	7.6	11.2	7	13.4	4.3	5.6	3.5	8.1	1.5
1981	8.2	12.3	7.2	14.2	4.8	6.2	3.6	8.6	1.7
1982	7.6	10.8	7.6	12.7	4.7	5.4	3.8	7.7	1.6
1983	11.7	17.1	10.9	19.3	7.4	8.6	5.4	11.7	2.6
1984	11.8	16.9	11.7	17.9	9.1	8.5	5.8	10.8	3.2
1985	13.7	19.7	13.4	20.7	10.6	9.9	6.6	12.6	3.7
1986	13.8	19.8	13.2	20.5	11	10	6.5	12.4	3.8
1987	12.6	18.2	12	18.6	10.3	9.1	6	11.2	3.6
1988	11.3	16.1	11.2	16.7	9.3	8.1	5.6	10.1	3.2
1989	9.8	14.1	9.4	14.6	7.7	7.1	4.7	8.9	2.7
1990	16.3	23.5	15.8	38.7	6.6	11.8	7.9	23.4	2.3
1991	16.4	22.8	17.3	23.4	14.9	11.5	8.6	14.2	5.2
1992	11.7	21.3	7.5	14.5	20.9	10.7	3.7	8.8	7.3
1993	13.3	17.3	17.1	18.5	12.9	8.7	8.5	11.2	4.5
1994	20	26.5	24.5	25.8	27.6	13.3	12.2	15.6	9.6
1995	19.5	26.7	21.5	25	27.2	13.4	10.7	15.2	9.5
1996	18.5	24.3	23	24.3	22.8	12.2	11.4	14.7	8
1997	10.5	14	12.4	13.9	12.3	7	6.2	8.4	4.3
1998	11.2	15	12.9	14.8	13.4	7.6	6.4	8.9	4.7
1999	9.2	12.7	10	12.6	9.7	6.4	5	7.6	3.4
2000	9.4	13.1	9.7	13	10.3	6.6	4.8	7.8	3.6
2001	10.4	14.4	11.3	13.8	12.3	7.2	5.6	8.3	4.3
2002	8.9	12.5	9.6	12	10.1	6.3	4.8	7.3	3.5
2003	8	11.7	7.5	11.1	8	5.9	3.7	6.7	2.8
2004	8.2	12	7.7	11.4	8.2	6	3.8	6.9	2.9
2005	7.5	10.8	7.3	10.6	7	5.4	3.7	6.4	2.4
2006	6.9	10.3	6	9.6	6.6	5.2	3	5.8	2.3
2007	6.6	9.4	6.5	9.1	6.5	4.7	3.3	5.5	2.3
2008	6.5	9.4	6.3	9.2	5.7	4.7	3.1	5.6	2

*Note: The data for fatality rates by sex or age groups were standardized to the Peruvian population of 2008.

**Table 5:** Crude and standardized average annual incidence, mortality, and fatality rates of RTIs in Peru in five-year time periods by sex and age group, 1973–2008.

		I	II	III	IV	V	VI	VII
		(1973–1977)	(1978–1982)	(1983–1987)	(1988–1992)	(1993–1997)	(1998–2002)	(2003–2008)*
**Incidence Rate**								
**Total Crude Incidence Rate**		135.4	149.0	113.4	72.0	79.7	124.5	162.4
**Standardized**	**Male**	95.6	101.9	80.0	49.3	61.0	91.3	114.9
	**Female**	39.7	47.1	33.3	22.6	18.7	33.1	47.5
	**≥18 years**	108.1	108.7	91.5	55.8	68.5	98.1	122.5
	**<18 years**	32.0	40.6	24.5	16.4	12.0	23.6	32.4
**Mortality Rate**								
**Total Crude Mortality Rate**		18.1	14.9	18.7	12.5	15.8	15.9	15.2
**Standardized**	**Male**	14.2	11.7	14.6	9.7	12.4	12.3	12.0
	**Female**	3.9	3.2	4.1	2.7	3.4	3.5	3.2
	**≥18 years**	18.2	14.6	17.7	11.4	13.9	13.0	12.3
	**<18 years**	2.1	1.8	2.3	1.6	2.2	2.6	2.2
**Fatality Rate**								
**Total Crude Fatality Rate**		13,6	10,0	16,5	17,1	21,3	12,8	9,5
**Standardized**	**Male**	7,6	5,8	9,2	9,8	10,9	6,8	5,3
	**Female**	5,0	3,4	6,1	6,1	9,8	5,3	3,4
	**≥18 years**	10,2	8,1	11,7	13,1	13,0	8,0	6,2
	**<18 years**	2,5	1,5	3,4	4,1	7,2	3,9	2,4

*Note: The latest five-year period includes 6 years. All rates per 100,000 inhabitants.

### Incidence

The highest total crude incidence rates were observed during the extremes of the study period, at 132 and 184 per 100,000 people in 1973 and 2008, respectively, while the lowest rates were observed in 1989 (Table 2). There was no evidence of variation in the IRR for either the total incidence or incidence within subgroups (Table 6). After adjusting for total population, vehicle density, and road density, however, we detected a notable increase of incidence with an IRR of 3.59 (95% CI 2.43–5.31). A similar trend was observed also among subgroups, with the greatest magnitude of change among females and those under age 18 (Table 6).

**Table 6:** Incidence rate ratios (IRR) and 95% confidence intervals (95% CI) of crude and adjusted incidence, mortality y fatality rates of RTIs in Peru, 1973–2008.

	Crude		Adjusted*		Covariates included in final model*
	IRR	95% CI	IRR	95% CI	
**Incidence**					
** Total**	1.00	0. 99–1.01	3.59	2.43–5.31	(1),(2) y (3)
** Male**	1.00	0.99: 1.01	3.07	2.07–4.55	(1),(2) y (3)
** Female**	1.00	0.99: 1.01	5.04	3,68–6.92	(1)
** ≥18 years**	1.00	0.99–1.01	2.55	1.77–3.78	(1),(2) y (3)
** <18 years**	0.99	0.98–1.00	6.87	4.30–10.99	(1),(3) y (4)
**Mortality**					
** Total**	1.00	0.99: 1.00	0.99	0.97–1.00	(2)
** Male**	1.00	0.99–1.00	0.98	0.97–0.99	(2)
** Female**	1.00	0.98–1.01	–	–	–
** ≥18 years**	0.99	0.98–1.00	0.98	0.97–0.99	(2)
** <18 years**	1.01	0.98–1.02	–	–	–
**Fatality**					
** Total**	1.00	0.99–1.01	0.26	0.15–0.43	(1)
** Male**	1.00	0.99–1.01	0.27	0.13–0.54	(1)
** Female**	1.00	0.99–1.01	0.20	0.08–0.46	(1)
** ≥18 years**	0.99	0.98–1.00	1.25	1.09–1.43	(1)
** <18 years**	1.01	1.00–1.03	0.26	0.14–0.48	(1)

*Notes: Variables tested in the adjusted models: (1) Total population, (2) Vehicle density, (3) Road density, (4) Allowance of imported used vehicles allowed. SOAT and the Zero Tolerance Plan were not associated with incidence, mortality or fatality in multivariable models.

The cubic spline curves that best fit the data had 5, 3, 5, 7 and 5 knots for the total incidence, those ≥18 years old, <18 years old, males y females, respectively. When fitting the models with splines, we observed the best fitting models had five knots for total incidence, three for those ≥18 years, five for those <18 years old, seven for males, and 5 knots for females.

**Figure 2:**
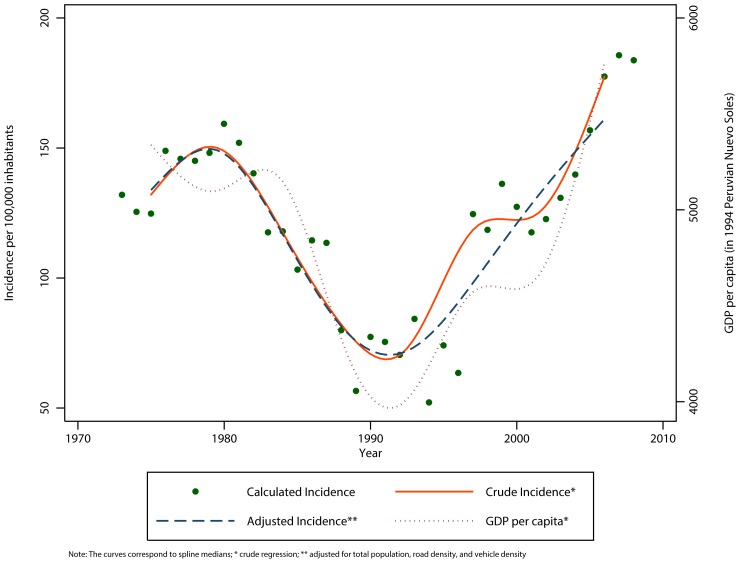
Total Crude* and Adjusted** incidence of road traffic incidences in Peru, 1973–2008; annual GDP per capita 1973–2008.

**Figure 3:**
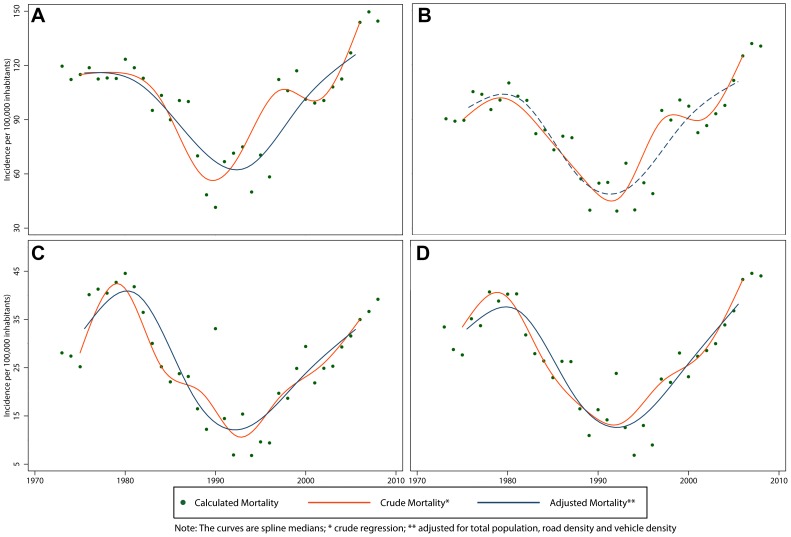
Crude* and adjusted** incidence of RTIs in Peru by age group and sex, 1973–2008. A) 18 years and older; B) Males; C) Less than 18 years; D) Females.

The total incidence of RTIs in Peru did not follow a linear trend (Figure 2). The adjusted curves suggest that the leveling observed in the crude incidence during the 1997–2000 time period can be explained by other variables. Notably, the behavior of the incidence rate over time is very similar to the GDP per capita trends. The total and subgroup incidence rate curves all show a peak during the 1980s, a decline in 1990 and, from there until 2008 a progressive increase (Figure 3). The regions whose incidence rates were greater than the national average in 2008 were Arequipa, Lima, Callao, Cuzco, Moquegua and Ucayali (Table 7).

**Table 7:** Incidence rates of RTIs in Peru by region, 1993–2008 per 100,000 inhabitants.

	1993	1994	1995	1996	1997	1998	1999	2000	2001	2002	2003	2004	2005	2006	2007	2008
**Perú**	**84.4**	**52.2**	**74**	**63.5**	**124.6**	**118.5**	**136.3**	**127.5**	**117.5**	**123**	**130.9**	**139.8**	**156.8**	**177.5**	**185.6**	**183.7**
**Amazonas**	30.3	12.9		11.2	10.2	44.0	34.5	112.6	107.9	75.7	60.6	34.8	51.9	56.5	57.8	89.3
**Ancash**	42.3	66.4	122.9	139.7	157.0	153.2	190.6	88.5	101.7	103.0	89.5	114.5	128.4	113.4	150.8	162.2
**Apurímac**	24.2	20.6	12.3	14.9	5.6	130.2	37.7	93.1	25.9	39.3	45.5	55.8	35.6	38.1	30.1	72.8
**Arequipa**	136.0	145.5	7.5	121.9	167.7	181.3	185.5	195.0	190.4	215.0	224.3	216.8	242.6	274.1	340.7	385.2
**Ayacucho**	16.2	25.8	166.5	31.3	18.7	17.0	52.7	190.5	129.8	75.5	88.4	96.2	81.5	110.8	95.2	117.5
**Cajamarca**	11.1	13.9	33.8	17.3	24.0	26.4	38.9	29.1	22.7	46.6	67.0	37.1	47.7	96.8	60.6	60.7
**Callao**	623.8	149.0	247.8	128.0	179.0	163.5	181.6	1274.2	194.6	155.0	128.6	154.1	155.8	204.6	206.1	254.4
**Cuzco**	87.5	58.6	18.2	63.2	137.8	139.2	107.1	100.5	87.2	86.5	66.6	98.4	114.5	171.1	176.6	204.8
**Huancavelica**	23.8	67.9	142.0	26.1	6.7	13.6	25.5	156.6	26.4	13.5	21.5	24.3	44.0	20.2	12.6	17.4
**Huánuco**	22.6	61.1	59.7	52.7	56.8	39.7	24.5	47.7	28.7	81.7	37.3	38.3	35.2	22.9	26.9	28.3
**Ica**	69.2	196.7	13.6	98.8	151.3	172.3	198.1	90.2	184.8	222.0	175.1	213.1	226.2	205.1	195.4	168.2
**Junín**	17.0	20.0	116.3	68.5	108.2	110.8	158.2	120.0	37.5	45.8	57.3	74.1	159.5	180.5	121.4	116.1
**La Libertad**	70.5	68.9	192.7	114.0	156.2	123.4	154.1	124.4	145.8	183.0	171.8	172.6	175.2	173.3	203.5	171.7
**Lambayeque**	67.2	27.0	124.0	147.6	34.3	63.5	47.8	132.8	75.1	111.0	303.1	111.9	98.4	94.5	96.1	103.5
**Lima**	120.0	45.3	51.1	29.3	215.4	188.4	230.8	78.0	190.9	188.0	198.8	243.0	281.3	319.7	337.9	311.0
**Loreto**	39.3	5.0	27.9	18.3	46.6	43.2	45.9	96.8	67.5	98.5	109.3	99.0	106.3	128.0	111.9	107.4
**Madre De Dios**	44.8	57.0	553.7	352.7	150.0	84.0	128.6	200.3	202.7	118.0	84.2	325.1	250.8	208.2	194.1	130.7
**Moquegua**	94.4	211.3	23.4	171.7	267.9	281.2	283.2	157.1	235.4	175.0	129.2	121.5	139.9	202.7	261.8	205.0
**Pasco**	24.3	7.5	93.8	105.3	79.1	131.3	88.1	76.9	76.0	24.2	38.0	24.5	14.1	13.1	35.5	17.6
**Piura**	8.5	36.8	93.1	114.0	41.8	27.9	45.2	39.4	35.4	53.8	49.8	54.9	55.6	58.4	60.1	59.2
**Puno**	26.7	13.2	7.9	28.8	78.5	33.2	47.0	60.4	39.0	32.8	27.7	30.9	44.5	67.4	77.2	64.2
**San Martin**	70.9	3.0	53.7	5.3	36.3	22.8	30.6	58.6	25.9	29.0	57.2	32.1	33.0	42.5	45.8	91.4
**Tacna**	148.6	203.5	20.0	241.9	228.0	235.9	215.1	180.3	240.8	214.0	189.4	198.9	96.0	37.5	55.8	56.2
**Tumbes**	85.7	35.6	73.3	70.1	72.5	67.9	100.7	195.7	156.9	107.0	75.0	80.5	114.5	137.1	137.9	123.9
**Ucayali**	34.0	29.9	27.7	19.3	50.5	121.8	97.1	95.4	91.6	74.3	80.6	117.1	74.6	74.5	132.3	192.2

### Mortality

The mortality rates per 100,000 people remained relatively constant from 1996 onward (Table 3) and in the last three five-year periods of the study (Table 5). The rates standardized by sex and age group indicate a larger concentration of mortality among men and those over age 18 (Table 5).

In the multivariable analysis, there was no evidence of observed variation in the temporal trends in the IRR in either the total mortality or by subgroups (Table 6), with all having an IRR slightly less than 1.00 (Table 6).

**Figure 4:**
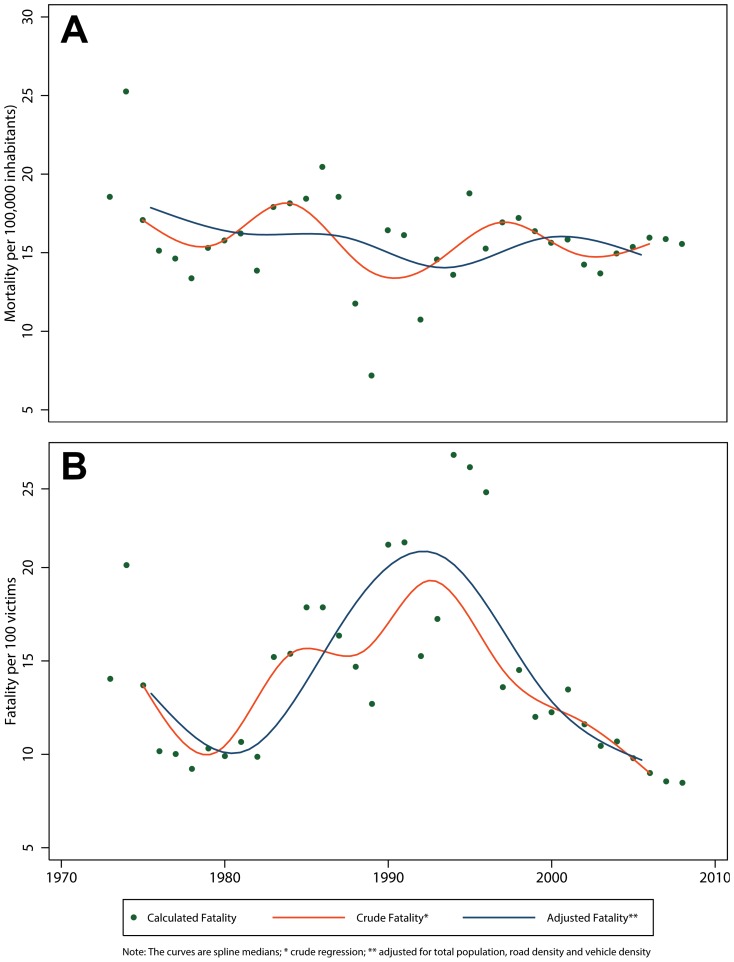
Total crude* and adjusted** mortality and fatality rates of RTIs in Peru, 1973–2008. A) Mortality; B) Fatality.

**Figure 5:**
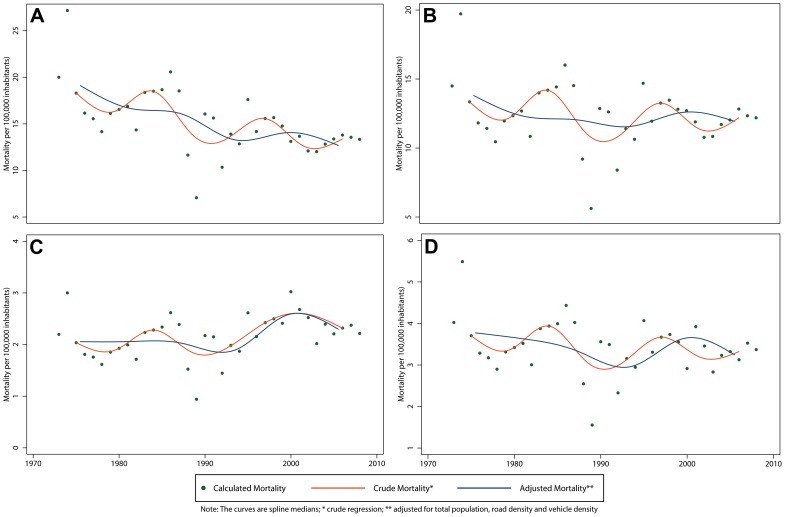
Crude and adjusted** mortality rate of RTIs in Peru by age group and sex, 1973–2008. A) 18 years and older; B) Males; C) Less than 18 years; D) Females.

The spline curve for total mortality rate that best modeled the data had 3 knots when adjusting for all factors related to mortality showing increasing and decreasing trends around each knot (Figure 4a). A similar pattern was observed in the mortality rate curves by sex, with a rate 3 to 5 times higher in males (Figure 5b) compared to females (Figure 5d). The 18 years and older group had a greater magnitude of mortality than the younger group and its trend followed a decreasing pattern over time (Figure 5a). For those under 18 years of age, we observed a slightly increasing trend in morality in the later years of the study period (Figure 5c). The regions of Madre de Dios, Cuzco and La Libertad had the greatest mortality rates during 2008, doubling the national average (Table 8).

**Table 8:** Mortality rate of RTIs in Peru by region, 1993–2008 per 100,000 inhabitants.

	1993	1994	1995	1996	1997	1998	1999	2000	2001	2002	2003	2004	2005	2006	2007	2008
**Perú**	**14.6**	**13.6**	**18.8**	**15.3**	**16.9**	**17.2**	**16.4**	**15.6**	**15.8**	**14.2**	**13.7**	**14.9**	**15.4**	**16**	**15.9**	**15.6**
**Amazonas**	3.0	7.1	-	5.9	1.4	2.9	6.4	15.7	21.0	16.7	6.6	10.3	10.1	23.3	16.7	24.6
**Ancash**	10.3	18.7	5.7	29.9	27.0	15.7	24.3	19.0	20.3	22.5	15.7	15.3	29.7	37.5	14.5	14.4
**Apurímac**	10.1	4.0	2.0	3.5	0.6	27.4	15.7	8.7	4.3	3.4	5.5	21.5	7.2	12.6	9.6	14.2
**Arequipa**	27.6	15.9	9.8	11.2	17.1	23.5	17.7	24.2	20.9	15.1	15.1	16.4	15.1	20.0	17.6	15.7
**Ayacucho**	2.9	4.7	11.1	2.3	1.8	2.2	11.1	7.3	19.7	7.8	10.0	7.5	16.1	10.1	1.7	31.0
**Cajamarca**	2.7	2.7	2.7	5.0	3.8	7.0	5.4	3.3	3.4	2.2	5.6	5.9	3.4	6.0	5.7	5.0
**Callao**	31.5	29.9	51.1	18.0	23.2	21.9	9.0	41.6	23.2	11.9	9.6	8.5	15.3	30.6	8.0	11.4
**Cuzco**	16.6	9.3	7.7	11.5	20.7	32.3	26.2	27.3	28.7	27.7	30.8	50.7	30.2	41.1	36.3	35.3
**Huancavelica**	4.4	70.3	16.0	4.1	0.9	2.8	4.6	24.5	9.6	2.9	11.8	11.3	26.7	8.5	9.1	3.4
**Huánuco**	6.0	22.5	19.1	16.9	21.3	11.2	4.9	16.0	3.9	13.8	6.2	7.0	3.1	4.3	4.2	4.7
**Ica**	9.4	21.0	17.7	11.6	20.2	25.7	16.3	16.2	40.6	35.7	18.4	26.6	18.2	20.8	21.7	17.0
**Junín**	4.2	7.4	81.1	13.8	39.9	26.6	41.7	25.7	13.6	12.0	10.9	12.8	21.2	16.1	15.8	9.9
**La Libertad**	8.5	14.2	16.6	19.8	27.7	26.5	43.2	31.7	32.7	40.7	43.5	29.3	33.7	27.9	24.7	28.2
**Lambayeque**	9.6	9.4	11.5	11.3	6.8	5.0	7.5	20.4	15.1	16.9	14.4	12.6	11.2	10.7	14.0	17.8
**Lima**	26.6	17.8	21.3	22.3	21.3	21.9	17.3	9.9	14.0	11.2	11.1	13.8	16.3	15.0	19.3	15.0
**Loreto**	10.1	4.2	8.9	4.9	0.8	2.6	2.1	8.0	3.2	3.9	7.1	2.8	4.3	2.9	7.2	3.3
**Madre De Dios**	17.7	7.4	12.0	10.5	6.8	13.3	7.1	27.7	2.7	45.8	20.4	29.7	24.1	4.7	9.2	56.3
**Moquegua**	6.6	19.2	28.7	41.4	23.2	46.8	17.7	14.7	20.3	10.0	19.5	7.1	45.9	25.1	15.7	15.4
**Pasco**	3.6	2.7	1.3	1.6	21.7	34.8	21.7	19.4	25.1	3.9	9.6	15.0	4.6	0.9	3.1	4.7
**Piura**	2.4	7.5	19.3	21.8	10.1	5.7	8.1	7.1	8.4	6.8	8.9	10.1	5.3	4.8	6.0	6.2
**Puno**	5.4	6.7	10.2	6.0	17.8	6.2	10.3	17.1	22.1	17.5	13.0	9.5	10.7	17.1	29.3	21.2
**San Martin**	9.6	1.8	0.8	1.4	2.3	3.7	4.0	11.4	5.1	8.1	14.2	5.3	5.6	6.2	9.3	24.1
**Tacna**	15.2	26.7	16.6	39.1	17.4	21.2	20.1	21.9	22.7	25.6	12.5	13.4	13.1	15.2	19.9	14.9
**Tumbes**	12.1	7.6	5.8	6.0	15.3	12.0	5.5	10.1	13.2	10.3	10.7	12.9	11.5	7.7	8.1	10.2
**Ucayali**	8.2	5.9	3.6	2.5	3.4	22.4	8.7	4.1	6.3	3.1	4.8	20.7	1.9	1.4	2.7	11.4

### Fatality

The highest fatality rates were in 1994, 1995, and 1996 when 20%, 19.5%, and 18.5% of the victims died (Table 4). The lowest rates were during the last three years (2006–2008) of the study period when less than 7% of the victims of RTIs died. Of the 7 five-year periods studied, the average fatality was highest during the 3^rd^, 4^th^, and 5^th^ five-year periods, corresponding to the years 1983-1997 (Table 5).

**Figure 6:**
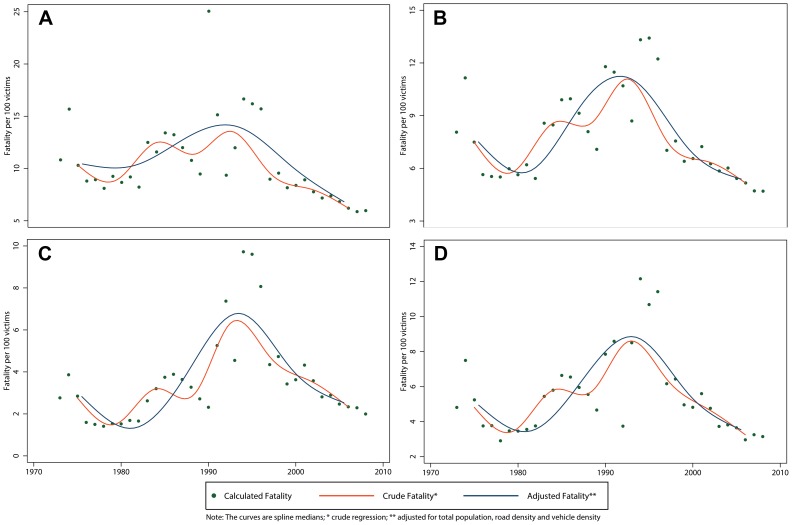
Crude and adjusted** fatality rate of RTIs in Peru by age group and sex, 1973–2008. A) Males; B) Females; C) 18 Years and Older; D) Less than 18 Years.

We found no significant variation in the IRR of the crude total fatality rate. When adjusting for the total population, nevertheless, we estimated a decrease with an IRR of 0.26 (95% CI 0.l5–0.43) (Table 6). The exception to this decreasing fatality trend were the subpopulation of adults 18 years and older who experienced a 25% increase over the study period (IRR 1.25, 95% CI 1.09–1.43). The best fitted spline curve had 5 knots for the total fatality rate (Figure 4b) as well as among subgroups except for those under 18, which required 6 knots (Figure 6). The total fatality rate followed a curvilinear pattern, suggesting a flattening of the fatality rate during the 1985–1990 time period. This trend could be explained by other variables defined *a priori*. In general, there was a peak in fatality in 1990 and a decrease from there onward.

The regions of Amazonas, Ancash, Cuzco, Huancavelica, Huánuco, Ica, Junín, Piura, Puno and San Martin showed a recurring higher fatality rate than the national average (Table 9). During 2008, the regions with the highest estimated fatality rate were Madre de Dios, Puno, Amazonas, Pasco, Tacna, Ayacucho and San Martin (Table 9).

**Table 9:** Fatality rate of RTIs in Peru by region, 1993–2008 per 100,000 RTI victims.

	1993	1994	1995	1996	1997	1998	1999	2000	2001	2002	2003	2004	2005	2006	2007	2008
**Perú**	**17.3**	**26**	**25.4**	**24**	**13.6**	**14.5**	**12**	**12.3**	**13.5**	**11.6**	**10.5**	**10.7**	**9.8**	**9**	**8.6**	**8.5**
**Amazonas**	10.0	54.9		52.6	13.3	6.7	18.6	14.0	19.5	22.1	10.8	29.5	19.4	41.2	29.0	27.6
**Ancash**	24.4	28.2	4.6	21.4	17.2	10.3	12.8	21.5	20.0	21.9	17.6	13.4	23.2	33.0	9.6	8.9
**Apurímac**	41.5	19.5	16.3	23.4	11.3	21.0	41.5	9.4	16.5	8.6	12.1	38.5	20.3	33.2	31.9	19.6
**Arequipa**	20.3	11.0	100.0	9.2	10.2	13.0	9.6	12.4	11.0	7.0	6.7	7.6	6.2	7.3	5.2	4.1
**Ayacucho**	17.8	18.1	6.6	7.2	9.4	13.1	21.0	3.8	15.2	10.3	11.3	7.8	19.7	9.1	1.8	26.4
**Cajamarca**	24.1	19.7	8.0	29.1	15.9	26.3	13.9	11.3	15.1	4.7	8.3	16.0	7.0	6.2	9.5	8.2
**Callao**	5.1	20.1	20.6	14.1	12.9	13.4	5.0	3.3	11.9	7.7	7.5	5.5	9.8	15.0	3.9	4.5
**Cuzco**	19.0	15.9	42.5	18.3	15.0	23.2	24.5	27.2	32.9	32.1	46.3	51.6	26.4	24.0	20.6	17.2
**Huancavelica**	18.3	79.7	11.3	15.6	13.9	20.5	18.1	15.7	36.2	21.7	54.9	46.4	60.6	42.0	72.5	19.7
**Huánuco**	26.6	36.8	32.0	32.0	37.5	28.3	19.8	33.5	13.6	16.9	16.5	18.3	8.8	18.6	15.6	16.6
**Ica**	13.6	10.7	100.0	11.7	13.3	14.9	8.2	18.0	21.9	16.1	10.5	12.5	8.1	10.1	11.1	10.1
**Junín**	24.8	36.8	69.8	20.1	36.9	24.0	26.4	21.4	36.3	26.2	19.1	17.3	13.3	8.9	13.0	8.5
**La Libertad**	12.0	20.6	8.6	17.4	17.7	21.5	28.0	25.5	22.4	22.2	25.3	16.9	19.2	16.1	12.1	16.4
**Lambayeque**	14.3	34.7	9.3	7.7	19.9	7.9	15.6	15.3	20.1	15.3	4.7	11.3	11.4	11.4	14.5	17.2
**Lima**	22.1	39.2	41.6	76.0	9.9	11.6	7.5	12.7	7.4	6.0	5.6	5.7	5.8	4.7	5.7	4.8
**Loreto**	25.6	84.1	32.0	26.7	1.7	6.1	4.6	8.2	4.7	3.9	6.5	2.8	4.0	2.2	6.5	3.1
**Madre De Dios**	39.6	13.0	2.2	3.0	4.6	15.8	5.6	13.8	1.3	38.9	24.2	9.1	9.6	2.3	4.7	43.0
**Moquegua**	6.9	9.1	94.1	24.1	8.7	16.7	6.2	9.4	8.6	5.7	15.1	5.9	32.8	12.4	6.0	7.5
**Pasco**	14.9	35.5	1.4	1.5	27.5	26.5	24.6	25.2	33.0	16.3	25.2	61.2	32.5	6.8	8.7	27.0
**Piura**	27.6	20.5	20.7	19.1	24.3	20.5	17.8	18.1	23.7	12.7	17.9	18.4	9.6	8.2	10.0	10.4
**Puno**	20.1	51.0	100.0	20.9	22.7	18.6	21.9	28.3	56.7	53.3	46.9	30.8	24.0	25.4	37.9	33.1
**San Martin**	13.6	60.7	1.4	26.8	6.4	16.4	13.1	19.4	19.5	27.8	24.9	16.6	17.0	14.5	20.3	26.3
**Tacna**	10.2	13.1	83.0	16.2	7.6	9.0	9.3	12.1	9.4	12.0	6.6	6.8	13.6	40.5	35.6	26.5
**Tumbes**	14.1	21.3	7.8	8.5	21.2	17.7	5.5	5.2	8.4	9.6	14.3	16.1	10.0	5.6	5.9	8.2
**Ucayali**	24.1	19.6	13.2	12.8	6.8	18.4	9.0	4.3	6.9	4.2	6.0	17.7	2.6	1.8	2.1	6.0

## Discussion

During the 35 years studied, there were nearly one million victims of RTIs reported by the police, demonstrating the magnitude of this public health problem in Peru. Our findings indicate that the incidence of RTIs increased year-to-year and overall that there was a nearly four-fold increase, however, this same trend was not observed for the mortality or fatality rates. This approach could be of use in other similar low- and middle-income settings using police-reported data to inform about the local challenges posed by RTIs.

The overall increasing trend in incidence was associated with the total population, vehicle density and road density. It is not surprising that these factors are related to incidence since they may be proxies for the exposure of the population to motor vehicle collisions. There were notably distinct trends among some of the subpopulations examined. Both women and children had much higher increase in incidence rates than men or adults, respectively. Additionally, adults' fatality rates increased rather than decreasing as for the general population. While these data did not allow us to study the causes of these trends in subpopulations specifically, they could be due to an increasing danger to pedestrians in Peru. As motorization increases, as we observed over the period of study, road traffic incident victimization increases, especially among vulnerable road users such as pedestrians [24]. This hypothesis may be supported by statistics reported in a descriptive study of police-reported collisions in Lima in 2008 where nearly 70% of pedestrians struck by motor vehicles are over age 18 and nearly half were female [46]. Further research is merited to understand the specific causes of these trends among these groups.

The curvilinear patterns for incidence and fatality rates that we observed using cubic splines demonstrate an interesting relationship that may be related to the financial crisis suffered by Peru in the late 1980s and early 1990s. This crisis may have affected these rates in two ways. First, when we superimposed the GDP curves, we observed coincidental rises and falls in the incidence curve, suggesting, as has been described in other studies [18], [47]–[49], that during financial crises RTIs decrease and in times of rapid economic growth they increase. The second effect of this crisis, however, may have resulted in a decreased reporting of RTI victims. This also may explain the fatality rates bell shape curve with the maximum reached at the peak of the crisis if the reporting of fatal collisions was not affected as greatly by the crisis. Nonetheless, the literature indicates also that during times of economic growth RTIs initially increase and later decrease at around a GDP per capita of $8,600 [50]. Many factors likely contribute to this shift in fatalities, including improved road infrastructure, safer vehicles, more resources for enforcement of traffic laws, better road user education, among others. This observation presents a potential challenge for Peru as it approaches this level of development.

The fairly constant adjusted mortality rate offers an interesting contrast to the incident and fatality rates. It was only associated with vehicle density in multivariable models, which may reflect the relationship between motorization and road fatality.[24] The fact that we did not observe any great changes in the RTI mortality rate and the decreasing fatality rate could certainly both be interpreted positively since incidence increased, but there are some potential issues with that perspective. The most important one is how road fatalities were reported during the time period. As described in the Methods, the police typically report a fatality if it occurred at the site of an incident rather than if an injured victim died later due to the RTI. While we adjusted for this underreporting as described in the Methods, that adjustment may not have been sufficient for the degree of underreporting that may exist in Peru. If mortality is much higher than what was observed and adjusted for, then Peru's road safety status may be worse than these results indicate. Another factor to consider is that the reporting of road fatalities may have been consistent over time while the incidence was not due to the seriousness of a RTI that results in a death at the scene of the incident. This phenomenon could also contribute to the decreasing fatality rate and constant mortality rate.

### Study limitations

The most important limitation of these data is that they are aggregate. Aggregate data limit the ability to complete detailed analyses between groups or other categories of interest. It also limited our ability to adjust for other factors that may be related to the probability of death. We attempted to overcome some of the limitations of these data by adjusting for total population, road density and vehicle density which can potentially modify the probability of the occurrence of RTIs. There are also, however, victim, vehicle, and environmental factors that, together with opportunity and quality of care, can influence the fatality rate. These factors are difficult to document and measure, and for example, adequate human resources [51] and insurance coverage for RTIs [52]remain a challenge to be addressed in the Peruvian setting.

We recognize that data reported by the police underreport RTIs, and the magnitude of this is impossible to determine with certainty, and there are known discrepancies between the volume of those affected reported by this institution when compared, for example, to the data of the *Asociación Peruana de Empresas de Seguros* (Peruvian Association of Insurance Companies). The period in which underreporting could be most notable was in the late 1980s when Peru suffered from an internal conflict with some zones controlled by the military without other state entities present. This situation could explain the fall of the study's indicators during this decade until the beginning of the 1990s. As mentioned before for other types of underreporting, it is difficult to determine the magnitude of this effect.

Considering these limitations and the need for timely and accurate health statistics, there is a great need to improve the collection of road traffic incident data in Peru [23]. While the data available allow for an assessment of the overall, crude trends, much work remains to be able to provide detailed, corrected statistics that can provide better evidence for policies and assessment.

### Conclusions

This study provides the first estimation of the epidemiological profile of RTIs in Peru by calculating trends over a 35-year period. As such, it constitutes a needed exercise that could be improved in terms of appropriately describing the Peruvian context. While overall incidence increased, children were clearly a subpopulation that had much higher incidence and fatality rates than other groups. Interventions that address this public health challenge should focus on children's play in the streets, as pedestrians walking to school and as unrestrained or inappropriately restrained passengers in motor vehicles. Overall, our approach, despite its limitations, can be of use in other similar low- and middle-income settings to inform about the local population-level challenges posed by RTIs. Being able to provide an accurate epidemiological profile of road injuries can illuminate the burden these injuries have on a nation's health, as well focus the appropriate resources to promote safer roads.

## Acknoweldgments

## References

[1] World Health Organization (2009) Global status report on road safety [Informe sobre la situación mundial de la seguridad vial]. Geneva: WHO.

[2] World Health Organization (2004) World report on road traffic injury prevention. Geneva: WHO.

[3] Sharma BR (2008) Road traffic injuries: a major global public health crisis. Public Health 122: 1399–1406. 18950819 10.1016/j.puhe.2008.06.009

[4] Instituto Nacional de Salud (2007) Prioridades de investigación en salud en el Perú: análisis del proceso. Lima: INS. Available: http://www.ins.gob.pe/insvirtual/ins/investigacionEnSalud/PrioridadesInvestigacion/libroPrioridadesInvestigacionINS.asp?fpp=1.

[5] Donroe J, Tincopa M, Gilman RH, Brugge D, Moore DA (2008) Pedestrian road traffic injuries in urban peruvian children and adolescents: case control analyses of personal and environmental risk factors. PLoS ONE 3: e3166. 18781206 10.1371/journal.pone.0003166 PMC2528934

[6] Secretaria Técnica del Consejo de Transporte de Lima y Callao (2008) Relación de puntos negros de accidentes de tránsito en el área de 36 distritos de Lima y Callao. Lima: CTLC–ST.

[7] Huicho L, Miranda JJ, Luna D, López L, Paca A, et al.. (2009) Estudio CAP, nivel de sensibilización, movilización, participación y fortalecimiento de organizaciones comunitarias en la prevención de daños y riesgos relacionados a accidentes de tránsito/Encuesta en padres e hijos [Informe Técnico]. Lima: Instituto Nacional de Salud, Salud sin Límites Perú.

[8] Bambaren C (2004) Características epidemiológicas y económicas de los casos de accidentes de tránsito atendidos en el Hospital Nacional Cayetano Heredia. Rev Med Hered 15: 30–36.

[9] Quistberg DA, Koepsell TD, Johnston BD, Boyle LN, Miranda JJ, et al.. (2013) Bus stops and pedestrian–motor vehicle collisions in Lima, Peru: a matched case–control study. Injury Prevention. 10.1136/injuryprev-2013-041023 PMC4321968 24357516

[10] World Health Organization (2009) Global status report on road safety: time for action. Geneva: World Health Organization.

[11] Institute for Health Metrics and Evaluation (2013) Global Burden of Disease Profile: Peru. Accessed February 24..

[12] World Health Organization (2006) Road Traffic Injury Prevention: Training Manual. Geneva: WHO. Available: http://www.who.int/violence_injury_prevention/road_traffic/activities/training_manuals/en/index.html.

[13] Rojas Medina Y, Espitia-Hardeman V, Dellinger AM, Loayza M, Leiva R, et al (2011) A road traffic injury surveillance system using combined data sources in Peru. Rev Panam Salud Publica 29: 191–197. 21484019

[14] Murray CJ (2007) Towards good practice for health statistics: lessons from the Millennium Development Goal health indicators. Lancet 369: 862–873. 17350457 10.1016/S0140-6736(07)60415-2 PMC7137868

[15] AbouZahr C, Adjei S, Kanchanachitra C (2007) From data to policy: good practices and cautionary tales. Lancet 369: 1039–1046. 17382830 10.1016/S0140-6736(07)60463-2

[16] Walker N, Bryce J, Black RE (2007) Interpreting health statistics for policymaking: the story behind the headlines. Lancet 369: 956–963. 17368157 10.1016/S0140-6736(07)60454-1

[17] Boerma JT, Stansfield SK (2007) Health statistics now: are we making the right investments? Lancet 369: 779–786. 17336655 10.1016/S0140-6736(07)60364-X

[18] Scuffham PA, Langley JD (2002) A model of traffic crashes in New Zealand. Accid Anal Prev 34: 673–687. 12214962 10.1016/s0001-4575(01)00067-7

[19] Grundy C, Steinbach R, Edwards P, Green J, Armstrong B, et al (2009) Effect of 20 mph traffic speed zones on road injuries in London, 1986–2006: controlled interrupted time series analysis. BMJ 339: b4469. 20007666 10.1136/bmj.b4469 PMC2791801

[20] Chen HY, Senserrick T, Chang HY, Ivers RQ, Martiniuk AL, et al (2010) Road crash trends for young drivers in New South Wales, Australia, from 1997 to 2007. Traffic Inj Prev 11: 8–15. 20146138 10.1080/15389580903434207

[21] Rahimi-Movaghar V, Zarei MR, Saadat S, Rasouli MR, Nouri M (2009) Road traffic crashes in Iran from 1997 to 2007. Int J Inj Contr Saf Promot 16: 179–181. 19941217 10.1080/17457300903024277

[22] Patton GC, Coffey C, Sawyer SM, Viner RM, Haller DM, et al (2009) Global patterns of mortality in young people: a systematic analysis of population health data. Lancet 374: 881–892. 19748397 10.1016/S0140-6736(09)60741-8

[23] Miranda JJ, Paca-Palao A, Najarro L, Rosales-Mayor E, Luna D, et al (2010) Evaluación situacional, estructura, dinámica y monitoreo de los sistemas de información en accidentes de tránsito en el Perú – 2009. [Assessment of the structure, dynamics and monitoring of information systems for road traffic injuries in Peru – 2009]. Rev Peru Med Exp Salud Publica 27: 273–287. 21072482 10.1590/s1726-46342010000200018

[24] Mohan D (2002) Road safety in less-motorized environments: future concerns. International Journal of Epidemiology 31: 527–532. 12055145 10.1093/ije/31.3.527

[25] Kopits E, Cropper M (2005) Traffic fatalities and economic growth. Accident Analysis & Prevention 37: 169–178. 15607288 10.1016/j.aap.2004.04.006

[26] Instituto Nacional de Estadística e Informática Capítulo 10: Violencia y Seguridad Pública. Compendio Sociodemográfico 1997–1998. Lima: INEI. Available: http://www1.inei.gob.pe/biblioineipub/bancopub/Est/Lib0065/cap10.htm.

[27] Centro de Investigación y de Asesoría del Transporte Terrestre Estadísticas: Accidentes a Nivel Nacional 1973–2006. Lima: CIDATT. Available: http://www.cidatt.com.pe/Accidentes%20a%20Nivel%20Nacional-2006.xls. Accessed: 17 Mar 2011.

[28] Centro de Investigación y de Asesoría del Transporte Terrestre Estadísticas: Accidentes a Nivel Nacional 1980–2008. Lima: CIDATT. Available: http://www.cidatt.com.pe/Accidentes%20a%20Nivel%20Nacional-2008.xls. Accessed: 17 Mar 2011.

[29] Dirección Técnica Demográfica, Instituto Nacional de Estadística e Informática (2002) Boletín Especial Nro 15 - Perú: Estimaciones y proyecciones de población total, urbana y rural por años calendario y edades simples, 1970–2025. Lima: INEI. Available: http://www1.inei.gob.pe/biblioineipub/bancopub/Est/Lib0503/Libro.pdf.

[30] Dirección Técnica Demográfica, Instituto Nacional de Estadística e Informática (2001) Perú: Población Total: 1950–2050. Lima: INEI.

[31] Instituto Nacional de Estadística e Informática (2007) XI Censo Población y VI de Vivienda. Lima: INEI. Available: http://censos.inei.gob.pe/censos2007/PagCensos_Queescenso.asp. Accessed: 17 Mar 2011.

[32] Instituto Nacional de Estadística e Informática (2009) Sistema de consulta de datos, XI Censo Población y VI de Vivienda. Lima: INEI.

[33] Jacobs G, Thomas AA, Astrop A (2000) Estimating global road fatalities (TRL Report 445). Crowthorne: Transport Research Laboratory. Available: http://www.transport-links.org/transport_links/filearea/publications/1_329_TRL445.pdf. Accessed: 17 Mar 2011.

[34] Planzer R (2005) La seguridad vial en la región de América Latina y el Caribe. Situación actual y desafíos. Santiago de Chile: CEPAL. Available: http://www.eclac.org/publicaciones/xml/3/23223/lcl2402e.pdf.

[35] Instituto Nacional de Estadística e Informática (2008) Perú: Compendio estadístico Lima: Ministerio Público, Fiscalia de la Nación. Available: http://www.mpfn.gob.pe/CD/compendio_estadistico/cap03/cap03.htm.

[36] Vásquez Cordano A, Bendezú Medina L (2008) Ensayos sobre el Rol de la infraestructura vial en el crecimiento económico del Perú. Lima: Consorcio de Investigación Económica y Social.

[37] Banco Central de Reserva del Perú Consultas a Series Estadísticas del BCRP. Lima: BCRP. Available: http://estadisticas.bcrp.gob.pe/index.asp?sFrecuencia=A.

[38] Cameron AC, Trivedi PK (1998) Regression analysis of count data. Econometric Society Monograph, No. 30. New York, NY: Cambridge University Press.

[39] Davies AR, Smeeth L, Grundy EM (2007) Contribution of changes in incidence and mortality to trends in the prevalence of coronary heart disease in the UK: 1996 2005. Eur Heart J 28: 2142–2147. 17636307 10.1093/eurheartj/ehm272

[40] Puiga X, Ginebra J, Gispert R (2005) Análisis de la evolución temporal de la mortalidad mediante modelos lineales generalizados. Gac Sanit 19: 481–485. 16483528 10.1016/s0213-9111(05)71401-1

[41] World Health Organization (2009) Global status report on road safety. Statiscal Anex Geneva, Switzerland: WHO. Available: http://www.who.int/violence_injury_prevention/road_safety_status/report/statistical_annexes_es.pdf.

[42] Statistical Consulting Group Stata Annotated Output: Negative Binomial Regression. Los Angeles, CA: UCLA, Academic Technology Services. Available: http://www.ats.ucla.edu/stat/Stata/output/stata_nbreg_output.htm. Accessed: 17 Mar 2011

[43] National Cancer Institute SEER*Stat Tutorials: Calculating Age-adjusted Rates. Bethesda, MD: NCI. Available: http://www.seer.cancer.gov/seerstat/tutorials/aarates/definition.html. Accessed: 17 Mar 2011

[44] Cole TJ, Stanojevic S, Stocks J, Coates AL, Hankinson JL, et al (2009) Age- and size-related reference ranges: a case study of spirometry through childhood and adulthood. Stat Med 28: 880–898. 19065626 10.1002/sim.3504 PMC2798072

[45] Kuha J (2004) AIC and BIC: Comparisons of Assumptions and Performance. Sociological Methods Research 33: 188–229.

[46] Secretaría Técnica del Consejo de Transporte de Lima y Callao (2009) La Vulnerabilidad de los peatones en la vialidad del área metropolitana de Lima y Callao [The vulnerability of of pedestrians in the roadways of metropolitan area of Lima and Callao]. Lima, Peru: Ministerio de Transportes y Comunicaciones.

[47] Suriyawongpaisal P, Kanchanasut S (2003) Road traffic injuries in Thailand: trends, selected underlying determinants and status of intervention. Inj Control Saf Promot 10: 95–104. 12772492 10.1076/icsp.10.1.95.14110

[48] Stuckler D, Basu S, Suhrcke M, Coutts A, McKee M (2009) The public health effect of economic crises and alternative policy responses in Europe: an empirical analysis. Lancet 374: 315–323. 19589588 10.1016/S0140-6736(09)61124-7

[49] Law TH, Umar RS, Zulkaurnain S, Kulanthayan S (2005) Impact of the effect of economic crisis and the targeted motorcycle safety programme on motorcycle-related accidents, injuries and fatalities in Malaysia. Int J Inj Contr Saf Promot 12: 9–21. 15814371 10.1080/17457300512331339166

[50] Kopits E, Cropper M (2005) Traffic fatalities and economic growth. Accident Analysis & Prevention 37: 169–178. 15607288 10.1016/j.aap.2004.04.006

[51] Rosales-Mayor E, Miranda JJ, Lema C, Lopez L, Paca-Palao A, et al (2011) [Resources and capacity of emergency trauma care services in Peru]. Cad Saude Publica 27: 1837–1846. 21986611 10.1590/s0102-311x2011000900017

[52] Miranda JJ, Rosales-Mayor E, Gianella C, Paca-Palao A, Luna D, et al (2010) Cobertura de la Ley de Atención de Emergencia y del Seguro Obligatorio contra Accidentes de Tránsito (SOAT) [Coverage of the Emergency Health Care Law and the Compulsory Insurance against Road Traffic Crashes (SOAT)]. Rev Peru Med Exp Salud Publica 27: 179–186. 21072468 10.1590/s1726-46342010000200004

